# Type and Timing of Childhood Maltreatment and Severity of Shutdown Dissociation in Patients with Schizophrenia Spectrum Disorder

**DOI:** 10.1371/journal.pone.0127151

**Published:** 2015-05-19

**Authors:** Inga Schalinski, Martin H. Teicher

**Affiliations:** 1 Department of Psychology, Clinical Psychology, University of Konstanz, Konstanz, Germany; 2 Department of Psychiatry, Harvard Medical School, Boston, Massachusetts, United States of America; 3 Developmental Biopsychiatry Research Program, McLean Hospital, Belmont, Massachusetts, United States of America; Central Institute of Mental Health, GERMANY

## Abstract

Dissociation, particularly the shutting down of sensory, motor and speech systems, has been proposed to emerge in susceptible individuals as a defensive response to traumatic stress. In contrast, other individuals show signs of hyperarousal to acute threat. A key question is whether exposure to particular types of stressful events during specific stages of development can program an individual to have a strong dissociative response to subsequent stressors. Vulnerability to ongoing shutdown dissociation was assessed in 75 inpatients (46M/29F, *M* = 31±10 years old) with schizophrenia spectrum disorder and related to number of traumatic events experienced or witnessed during childhood or adulthood. The Maltreatment and Abuse Chronology of Exposure (MACE) scale was used to collect retrospective recall of exposure to ten types of maltreatment during each year of childhood. Severity of shutdown dissociation was related to number of childhood but not adult traumatic events. Random forest regression with conditional trees indicated that type and timing of childhood maltreatment could predictably account for 31% of the variance (*p* < 0.003) in shutdown dissociation, with peak vulnerability occurring at 13-14 years of age and with exposure to emotional neglect followed by various forms of emotional abuse. These findings suggest that there may be windows of vulnerability to the development of shutdown dissociation. Results support the hypothesis that experienced events are more important than witnessed events, but challenge the hypothesis that “life-threatening” events are a critical determinant.

## Introduction

Adverse childhood experiences are common in patients with severe psychopathology, including patients diagnosed with psychotic disorders e.g., [1,2]. Despite increasing interest in the relationship between childhood trauma and psychosis, relatively little attention has been paid to the importance of trauma-related symptoms such as dissociations. However, recent studies have started to actively explore the relationship between traumatic stress and dissociative symptoms in patients with psychotic disorders [3,4,5,6].

Overall, there is a strong relationship between exposure to adverse childhood experiences and presence of dissociative symptoms in adulthood [7,8], and recent etiological models acknowledge dissociative symptoms as a long-term consequence of traumatic stress [9]. The defense cascade model describes adaptive reactions to life-threat including signs of shutdown dissociation. When confronted with an imminent life-threat, for which flight or fight are no longer viable options to counter danger, the organism may choose immobility and dissociative responding [10,11]. Dissociative responses may thus be adaptive, enabling the organism to escape the threatening situation as well as the internal distress and emotional arousal and may persist as a long-term consequence [12,13,14,15,16]. However, ongoing dissociation affects perceptual, emotional and cognitive functions as well as motivation and interferes with an integrative representation of the environment and the self [9].

Traumatic experiences with a high proximity to danger such as sexual or physical abuse are associated with severe shutdown dissociation, whereas witnessing traumatic events appears to have no significant effect [17]. This is consistent with observations that threat to oneself has a greater effect on symptom severity [15] and heart rate reactivity [18] than witnessing traumatic events. However, prior studies have not necessarily considered the relative impact of witnessed versus self-experienced traumatic events from a developmental perspective. Whereas an adult possesses the strength to encounter danger or the power for flight or fight, a child is more likely to show peritraumatic and persistent dissociative responding [19,20,21]. Hence, one study reported greater effects on dissociative symptoms in adulthood of witnessing violence to siblings during childhood than actually experiencing physical abuse [22]. This underscores the potential importance of timing of early life stress on long-term consequences.

Childhood is thought to reflect a particularly sensitive developmental window for different brain regions in structure and function [23,24]. Adversities during this sensitive period may target brain structures undergoing rapid development, such as myelination, synaptic overproduction or synaptic pruning [25]. From the perspective of neurobiology, the infant brain is a plastic organ with period of increased sensitivity guided by constantly modified gene-environment interactions [26]. Therefore, brain regions that mature at different rates may have their own specific developmental windows of vulnerability when they are maximally susceptible to abuse or neglect. In a sample of patients with repeated episodes of sexual abuse it was reported that exposure between 3–5 years and 11–13 years were associated with reduced hippocampal volume, whereas volume of the prefrontal cortex was negatively affected by sexual abuse between 14–16 years [23]. Neurobiological studies have also shown that different types of maltreatment specifically target brain regions and pathways that convey the adverse experience. For example, witnessing domestic violence was associated with alterations in visual cortex [27] and visual-limbic pathway [28]. In contrast, exposure to high levels of parental verbal abuse were associated with alterations in auditory cortex [29] and auditory pathways [30], while childhood sexual abuse was associated with thinning of the genital representation area of somatosensory cortex in females [31].

It is also likely that the clinical consequences of childhood maltreatment may depend, at least in part, on type and timing of exposure [32]. While many studies have shown a dose-dependent relationship between composite severity of exposure and outcome, other studies have drawn attention to the deleterious effects of specific types of maltreatment.

Types of childhood adversities specifically associated with symptoms of dissociation may depend, to some degree, on the population being studied. Teicher et al. [33], in a community sample, reported high levels of dissociation in individuals with combined exposure to three types of abuse (physical, sexual and emotional) as well as those who both witnessed domestic violence and experienced parental verbal abuse [22]. Other studies, using community samples have also reported increasing risk for dissociative symptoms with exposure to emotional abuse and emotional neglect [8,34]. Emotional neglect showed the strongest association with dissociation in a sample of psychiatrically ill adolescents [35]. In patients with affective disorders, the severity of shutdown dissociation was highest in those with cumulative stressful experiences during adolescence, especially sexual and emotional abuse [36].

Studies examining subjects with schizophrenia or other psychotic disorders also highlight the importance of emotional abuse. For example, dissociation correlated most strongly with emotional abuse and secondarily with physical abuse in a sample of adults with schizophrenia [3]. Similarly, emotional abuse was the one form of maltreatment on the Childhood Trauma Questionnaire (CTQ) that was consistently associated with symptoms of dissociation from admission to stabilization in hospitalized women with schizophrenia spectrum disorder [5]. However, in a larger sample they found that sexual abuse best predicted severity of dissociation once patients were stabilized [37]. Emotional abuse was found to have the strongest relationship with dissociation in both chronically psychotic patients and in individuals experiencing their first psychotic episode [38].

Physical neglect has also emerged as a significant predictor in some studies including a male subsample in one report [38], a predominantly male schizophrenic sample in another [39] and in female schizophrenic sample at the time of admission but not after stabilization [5]. Şar and colleagues [4] found that physical neglect and physical abuse were the best predictors of dissociation in a stepwise linear regression, but all forms of maltreatment were increased to a similar statistical degree in high versus low dissociating subjects with schizophrenia [4]. In an early study Greenfield and colleagues [40] reported that dissociation scores were highest in first episode psychotic patients reporting combined physical and sexual abuse. However, the key factor was actually whether or not the perpetrator was a parent. Overall, studies in schizophrenic or psychotic samples show a clear association between exposure to childhood maltreatment and dissociation, but differ in their determination as to what types of maltreatment were most strongly predictive. Interestingly, emotional abuse, emotional neglect and physical neglect emerged as important predictors at least as frequently as physical abuse, and more frequently than sexual abuse.

While the relationship between maltreatment and severity of dissociation is strong in subjects with psychotic disorders, the association between maltreatment and severity of psychosis is less clear. On one hand, epidemiological studies provide evidence for a potential causal relationship between maltreatment and emergence of positive but not negative psychotic symptoms [41,42,43]. Types of maltreatment associated with positive psychotic symptomatology include: sexual abuse e.g., [42,44,45,46], emotional neglect [47] and peer victimization [48,49]. However, while maltreatment appears to increase risk for emergence of psychotic symptoms, some studies suggest that psychotic symptoms may be of comparable severity in schizophrenic subjects with and without histories of childhood maltreatment. For example, Greenfield et al. [40] found no difference in symptom severity between first episode patients with and without childhood abuse, and only a marginal increase in length of stay for the maltreated subjects. Similarly, Şar et al. [4] found no significant association between childhood maltreatment and severity of positive or negative symptoms in subjects with schizophrenia. However, Vogel et al. [50] reported that childhood trauma predicted negative symptoms in schizophrenic subjects. Further, an earlier age of onset, longer length of stay and greater frequency of relapse have been reported in chronically psychotic subjects with histories of maltreatment [1]. In short, more research is needed to determine in schizophrenic subjects whether exposure to particular types of abuse, during specific developmental stages, are associated with increased severity of positive and negative psychotic symptoms and symptoms of dissociation.

It is important to note that means of assessing dissociation in prior studies varied and could include anything from minor dissociative experiences to major-pathological dissociation, though most studies used the Dissociative Experience Scale (DES) [51]. The DES was created based on clinical perceptions of dissociative symptomatology, and was validated by showing higher DES scores in subjects with dissociative identity disorder than in other major psychiatric disorders. The DES is an important instrument and has played a pivotal role in the resurgence of interest in dissociation during the last three decades. However, with this resurgence has come an understanding that dissociation is a broad construct with a number of different meanings or interpretations.

Recently an important distinction has been made between the dissociative processes of compartmentalization and detachment [52,53,54]. Briefly, compartmentalization is defined as a failure to deliberately control volitional processes and often presents as an inability to bring usually accessible information into conscious awareness [54]. Clinically, compartmentalization is characterized by dissociative amnesia and conversion symptoms [54], and overlaps with the concept of “somatoform dissociation” [55,56,57].

Detachment, in contrast, is defined as the subjective experience of an altered state of consciousness and typically includes derealization (e.g., feeling that things are not real, that it is just a dream), depersonalization (e.g., out-of-body experiences) and a diminution of the emotional experience [54]. It is this dissociative process that is embraced by DSM-5 (Diagnostic and Statistical Manual of Mental Disorders 5^th^ version) in their recognition of a dissociative subtype of posttraumatic stress disorder (PTSD) [58].

The evolutionary model of the defense cascade by Schauer and Elbert [9] provides another perspective on dissociative processes. They propose that there is a coherent sequence of six fear responses (Freeze-Flight-Fight-Fright-Flag-Faint) that escalate as a function of defense possibilities and proximity to danger during life-threat. Peritraumatic dissociative symptoms emerge during the ‘Fright-Flag-Faint’ sequence, and these reactions may be replayed when the fear network is activated through internal or external triggers [9]. In the earlier stages of the cascade sympathetic responses dominate, but the later stages are characterized by increasing degrees of parasympathetic dominance. The Shutdown Dissociation Scale (Shut-D) [17], which is an interview-based measure derived from this model, focuses on the shutting down of sensory and motor functions and feelings of fainting, dizziness or nausea. Although they emerge from very different conceptual frameworks, there is substantial overlap between items on the Shut-D and items on the Somatoform Dissociation Questionnaire (SDQ-20) [56]. However, the SDQ-20 includes other items such as painful perceptions that do not fit within the defense cascade mode and the Shut-D includes a detachment-related item (out of body experience) that would not fit within the SDQ-20. In short, the concept of shutdown dissociation is based on an evolutionary perspective of physiological responses to escalating threats. It overlaps substantially with somatoform dissociation, partially with detachment, and with its emphasis on parasympathetic dominance, also aligns with components of polyvagal theory [59]. Given the strong, theoretical and trauma-based focus of this scale we felt that it would be an ideal instrument for assessing the presence of dissociative symptoms in participants with psychotic disorder and for delineating their relationship to early life traumatic and non-traumatic stressors.

In the present study we assessed traumatic experiences across the lifespan (childhood and adulthood) with particular emphasis on type and timing of childhood adversities. We hypothesized that a greater number of different traumatic event types, particularly during childhood, would be associated with more severe shutdown dissociation. Based on previous literature [3,4,36,38], we also assumed that there would be differential effects of timing and type of maltreatment on symptoms of shutdown dissociation. Hence, we sought to determine if there were windows of vulnerability across age for shutdown dissociation, and addressed the questions if the timing and type of maltreatment and abuse mattered in a sample of patients diagnosed with psychotic disorders. Further, we investigated whether there were maltreatment-related vulnerable windows for the expression of positive and negative symptoms of schizophrenia.

## Materials and Methods

### Ethics Statement

Prior to the interview, participants were informed about the goal of the study and each participant provided written informed consent. The capacity of the participants to provide informed consent was also approved by their treating psychologist or psychiatrists as well as confirmed during the interview. The project has been reviewed and approved by the Institutional Review Board (Ethics Committee) of the University of Konstanz.

### Settings

Experienced-psychologists carried out structured clinical interviews following stabilization from acute psychotic or manic symptoms. Participants were paid €10 and travel costs. We carried out 75 interviews between January 2013 and December 2013. Two patients refused to participate for the following reasons: One refused because the participant felt bothered by his childhood experiences and one participant refused to participate because of distrust.

### Instruments

The number of traumatic experiences was assessed with the sum of the event checklist [60]. Sum scores were calculated separately for traumatic experiences that were experienced up to the age 18 and in adulthood. We furthermore differentiated between the number of traumatic event types that were self-experienced (high proximity to danger) and the number of different traumatic event types that were witnessed (low proximity to danger). A traumatic event type was judged as self-experienced if the participant was the victim or as witnessed if the participant had observed someone else being seriously threatened.

The Maltreatment and Abuse Chronology of Exposure (MACE) Scale for adults interview version [61,62,63] was applied, that was specially developed to assess retrospective recall of exposure to ten different types of childhood maltreatment (i.e., parental physical and verbal abuse, parental non-verbal emotional abuse, familial and non-familial sexual abuse, witnessing interparental violence, witnessing abuse to siblings, peer verbal abuse and physical bullying, emotional and physical neglect) during each year of childhood up to age 18. The scale was developed using item response theory and found to have excellent test-retest reliability (*r* = 0.91, *n* = 75 at 10 weeks). The MACE MULTI score indicates the number of different types of childhood adversities experienced whereas the MACE SUM score indicates overall severity of exposure. MACE MULTI score correlated *r* = .71 (95% CI 0.68–0.74, df = 1043, *p* < 10^–16^) with the Adverse Childhood Experience score (ACE, [64]), while MACE SUM correlated *r* = .74 (95% CI 0.69–0.78, df = 395, *p* < 10^–16^) with the childhood trauma questionnaire (CTQ [65]) total score. However, MACE-MULTI and MACE-SUM accounted, on average, for 2-fold more of the variance in psychiatric symptom ratings (anxiety, depression, somatization, anger-hostility, dissociation, limbic irritability, suicidality) than ACE or CTQ based on variance decomposition.

To assess dissociative responding, we used the 13-item Shutdown Dissociation Scale (Shut-D; [17]) a structured interview to assess the severity for shutdown dissociation. The interviewer ask for the frequency of the following symptoms: fainting, dizziness/ transitory blindness, transitory deafness /changed acoustic perception, changed visual perception, numbness, transitory paralysis, analgesia, sudden feeling of heaviness and tiredness, tension, feeling of nausea, out-of-body, inability to speak, and weakness accompanied with hot flash. Responses to all items were given on a scale including 0 (not at all), 1 (once a week or less), 2 (2–4 times a week), to 3 (5 or more times a week). Summed scores can range from 0 to 39. When completing this interview, interviewers established the timeframe for which these shutdown dissociation symptoms were reported. The interviewer selected a timeframe within the past six months in order to acquire an overview of the patient’s suffering in their everyday life. The Shut-D appears to have sufficient internal reliability (Cronbach’s α = .88), and excellent retest reliability (*r* = .87), while the summed score of the scale reliably separated patients with exposure to trauma (in different diagnostic groups) from healthy controls. Furthermore, the scale shows high convergent validity with the sum score of the Dissociative Experiences Scale (*r* = .86, *p* < .001), a scale that has dominated in the research of dissociation [51]. The Positive and Negative Syndrome Scale (PANSS) was applied to assess the positive, negative and global symptom severity [66]. The level of functioning was described with the Global Assessment of Functioning score (GAF; [67])

### Participants, Demographical and Clinical Data

Seventy-five patients (*n* = 26 female) with a main diagnosis of schizophrenia spectrum disorders according to (International Classification of Mental and Behavioral Disorders Tenth Version (ICD-10): F20–F25, [68]) and mean age of *M* = 31 (*SD* = 10) years were recruited from the inpatient pool at the Zentrum für Psychiatrie Reichenau (see demographic and clinical data in Table 1). Data from *n* = 60 of the patient sample were reported in [69].

**Table 1:** Means and Standard Deviations of the Demographic Data, Trauma-related Characteristics as well as Symptom Severity and Functioning.

	*M/ n*	*SD/ %*
**Demographic Data**		
Total Sample	75	
Female	26	34.7%
Male	49	65.3%
Number of Patients (First Hospitalization)	29	38.8%
Age	31	10
Years of Education (in years)	13.5	3
**Trauma-related Characteristics**		
Severity of Childhood Adversities		
MACE SUM	25.8	15.3
MACE MULTI	2.3	2.1
Number of Different Traumatic Event Types in Childhood		
Self-experienced	1.6	1.7
Witnessed	0.6	0.8
Number of Different Traumatic Event Types in Adulthood		
Self-experienced	1.9	1.7
Witnessed	0.6	1
**Symptom Severity and Functioning**		
Symptom Severity of Shutdown Dissociation	3.5	4.5
Positive and Negative Syndrome Scale Total Score	67.3	14
Positive Symptom Severity	15	4.6
Negative Symptom Severity	17.4	6.1
Global Symptom Severity	34.9	6.8
Global Assessment of Functioning Score	48.4	15.3

*Note*. *M* = Mean, *SD* = Standard Deviation, *n* = number of patients

### Statistical Analysis

Analyses were performed using R version 2.15.1 (3.0.0) and SPSS 20.0; alpha level was set at. 05 (two-tailed). Pearson correlation coefficients and partial correlations were used to assess the association between exposure to experienced and witnessed traumatic events in childhood or adulthood and severity of shutdown dissociation. Linear mixed effects models were used to assess differences in severity of exposure to each type of maltreatment across childhood in subjects with the lowest levels of shutdown dissociation (lowest quartile, Shut-D Scale = 0 ± 0, *n* = 22) and those in the highest quartile (Shut-D Scale > 5, *M* = 9.65 ± 4.45, *n* = 20).

### Random Forest Regression with Conditional Trees

Overall, the MACE provides retrospective data on exposure to 10 types of maltreatment across 18 years of development. It would be ideal to know whether severity of exposure to a particular type of maltreatment at a specific age was an important risk factor or predictor of developing shutdown dissociation. Conventional analytical techniques such as multiple regression are not suitable for this task; the most critical reasons being that the number of potential predictor variables (m = 180) greatly exceeds the number of subjects (*n* = 76), and there is substantial collinearity (or multicollinearity) in degree of exposure to a particular type of maltreatment at adjacent ages.

This situation however is a common occurrence in data mining and ‘big data’ analytics and several techniques exist for identifying important predictor variables under these circumstances. A general strategy is to use predictive modeling in which a machine learning algorithm is trained to provide an accurate fit to a training set which is then evaluated for predictive validity on a separate test set. Once a good predictive model is established a variety of techniques exist for identifying the most important predictor variables within the model.

A particularly useful machine learning strategy is random forest regression, which used decision trees as the base learners [70]. Decision trees by themselves can often fit the data well but are typically weak predictors. Random forest regression improves predictive validity by creating a forest of decision trees. Trees within the forest differ from each other as they are each generated from a different subset of the data and each tree is constrained in the number of predictor variables it can consider at each decision point [70]. New data is run through each tree in the forest and the outcome of the trees averaged.

This counterintuitive strategy works well and generally provides predictive models that are superior to those produced using conventional regression techniques and are at least on par with other machine learning approaches such as neural networks and support vector machines [71]. In addition to high predictive accuracy, random forests can successfully identify important predictor variables in situations where number of predictors greatly exceeds number of subjects. Further, it does not require that the variables be normally distributed, they can be distributed or scaled in any way and it is resistant to multicollinearity [70]. Random forest regression is also advantageous as the tree structure allows for the detection and modeling of interactive effects between the variables.

Breiman [70] in developing this strategy also provided a novel means of determining variable importance. The importance of each predictor variable is assessed by randomly permuting each variable in turn and determining how much this degrades model fit. Permuting important predictor variables decreases fit to a large degree whereas permuting unimportant predictors has little or no impact.

We used a variant of Breiman’s approach with conditional trees as the base learner (‘*cforest* ‘in R package *party* [72]). This approach rectifies a potential problem with random forest regression that can inflate the importance of predictor variables with many versus few levels or categories. Random forest with conditional trees appears to provide an unbiased estimate of variable importance that is not influenced by number of categories, mean value, range, or variance of the predictor variables [72].

Training and testing were accomplished using 10 repetitions of 10-fold cross validation. Briefly, data are split into ten sets. Nine sets are used to train the model and the tenth set is used to test its predictive validity. The process is repeated 10 times so that each set is used once to test validity. This entire process is then repeated 10 times on different random splits of the data. Predictive validity and variable importance measures were averaged across the 10-fold cross validation and reliability derived from the ten random split repetitions. Data were limited to ages and types of maltreatment that were experienced by at least 5% of the sample, which reduced the number of predictor variables to 149. From the complete analysis results were collapsed across age or maltreatment type to indicate the maximal importance of exposure to each type of abuse regardless of age, and maximum sensitivity to maltreatment at each age regardless of type of exposure.

## Results

### Prevalence of Traumatic Experiences in Childhood and Adulthood and Correlates with Shutdown Dissociation

**Fig 1:**
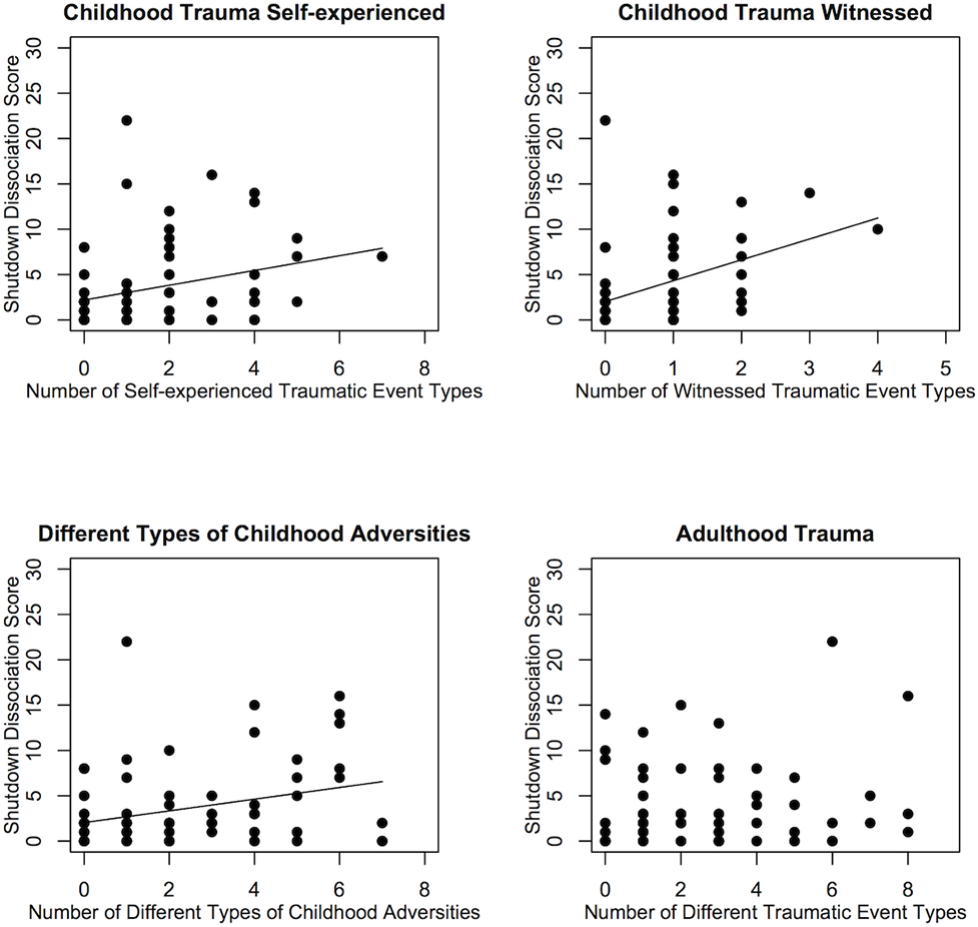
Dose-dependent Effects of Childhood and Adulthood Traumatic Experiences and Shutdown Dissociation. The dose-dependent effects of the number of different types of traumatic experiences in childhood and adulthood, as well as childhood adversities on shutdown dissociation are displayed.

Traumatic experiences in childhood were reported by 72% with personal life-threat and 60% witnessed at least one traumatic experience. Traumatic experiences up to the age 18 that were self-experienced as well as witnessed were correlated with the severity of shutdown (*r*_self_ = .30, *p* = .009; *r*_witnessed_ = .41, *p* < .001). Eighty-three percent experienced at least one trauma event type, and 53% witnessed at least one trauma type in adulthood. In contrast, the number of different traumatic event types in adulthood didn’t yield significant association with the severity of shutdown dissociation (*r*_self_ = .08, *p* = .478; *r*_witnessed_ = .10, *p* = .384; Fig 1).

### Prevalence of Childhood Adversities and Correlations with Traumatic Experiences and Shutdown Dissociation

Altogether, 72% fulfilled the criteria for one or more type/s of childhood abuse or maltreatment. Twenty-one percent were exposed to one type, 21% to 2–3 different types and 28% to 4–7 different types. On average, individuals reported having been exposed to *M* = 2.3 (*SD* = 2.1) different types of childhood adversities. There was a significant association between number of different types of childhood maltreatment reported on the MACE and number of additional traumatic events experienced, but not witnessed, in childhood (*r*_self_ = .25, *p* = .028; *r*_witnessed_ = .16, *p* = .166) and adulthood (*r*_self_ = .25, *p* = .033; *r*_witnessed_ = .15, *p* = .197). After controlling for the number of different traumatic experiences in adulthood and additional traumatic event types in childhood, the associations between the severity score of childhood adversities (MACE sum score) showed still significant correlation (*pr* = .32, *p* = .006).

### Differences in Type and Timing of Maltreatment

**Fig 2:**
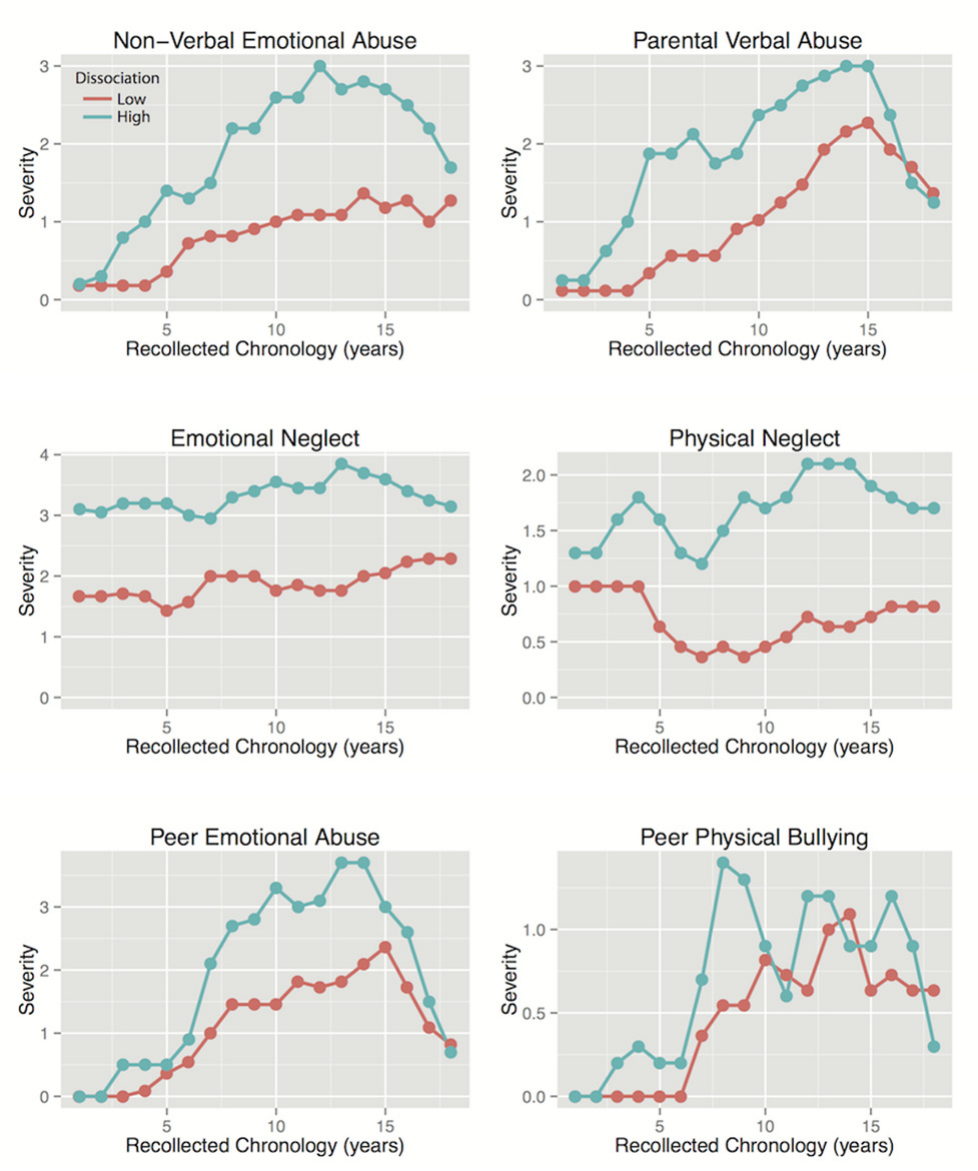
Severity of Childhood Adversities in Subjects with High versus Low Shutdown Dissociation. Severity of exposure to different forms of maltreatment across development in subjects with the lowest levels of shutdown dissociation and subjects with the highest levels.

**Fig 3:**
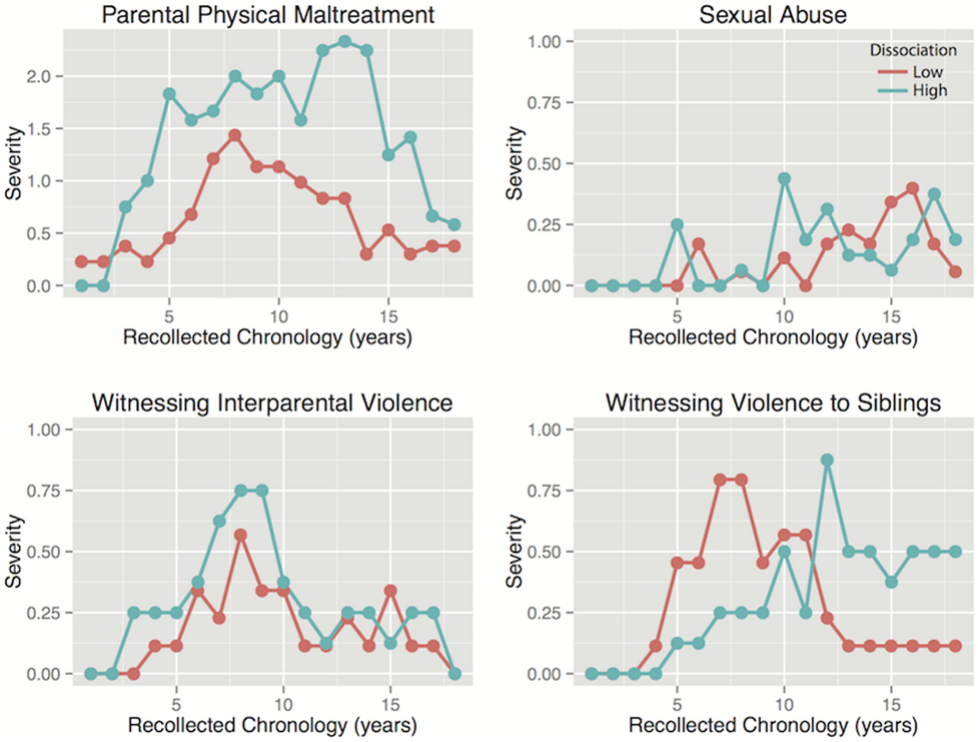
Severity of Childhood Adversities in Subjects with High versus Low Shutdown Dissociation. Severity of exposure to different forms of maltreatment across development in subjects with the lowest levels of shutdown dissociation and subjects with the highest levels.

Subjects in the lowest and highest quartiles for shutdown dissociation were compared for differences in reported exposure to each type of maltreatment across childhood using linear mixed effects models. As seen in Figs 2 and 3 as well as Table 2 there were significant differences between these subgroups in degree of exposure to physical and emotional neglect and parental verbal and non-verbal emotional abuse as well as parental physical maltreatment.

**Table 2:** Linear mixed effects models indicating main effects of recollected ages of exposure, low and high dissociation group and their interaction on severity of exposure scores.

Types of Exposure	Recollected Age		Dissociation Group		Recollected Age x Dissociation Group		Gender	
	F_17.~634_	% var.	F_1.~634_	% var.	F_17.~6349_	% var.	F_1.~634_	% var.
Emotional Neglect	0.87	0.26%	5.99^a^	0.11%	1,52	0.46%	0.04	0.00%
Parental Nonverbal Emotional Abuse	7.21^e^	3.68%	3.90^a^	0.12%	2.82^d^	1.44%	0.58	0.02%
Parental Physical Maltreatment	5.04^e^	6.04%	4.02^a^	0.28%	1.55	1.86%	5.41^a^	0.38%
Parental Verbal Abuse	4.13^e^	3.71%	5.68^a^	0.30%	1.10	1.00%	0.00	0.00%
Peer Emotional Abuse	8.65^f^	7.14%	2.16	0.10%	1.26	1.04%	1.37	0.07%
Peer Physical Bullying	2.83^d^	3.93%	2.60	0.21%	0.69	0.96%	3.47	0.28%
Physical Neglect	1.48	0.96%	7.33^b^	0.28%	1.12	0.73%	0.16	0.01%
Sexual Abuse	1.03	0.75%	0.31	0.03%	0.99	0.68%	0.22	0.02%
Witnessing Interparental Violence	2.20^b^	3.47%	0.39	0.04%	0.37	0.58%	1.05	0.10%
Witnessing Violence to Siblings	1.51	2.39%	0.22	0.02%	1.25	0.98%	1.92	0.18%

^a^p < 0.05,

^b^p < 0.01,

^c^p < 0.001,

^d^p < 10^–4^,

^e^p < 10^–6^,

^f^p < 10^–16^

### Importance of Type and Timing from Predictive Modeling and Machine Learning

**Fig 4:**
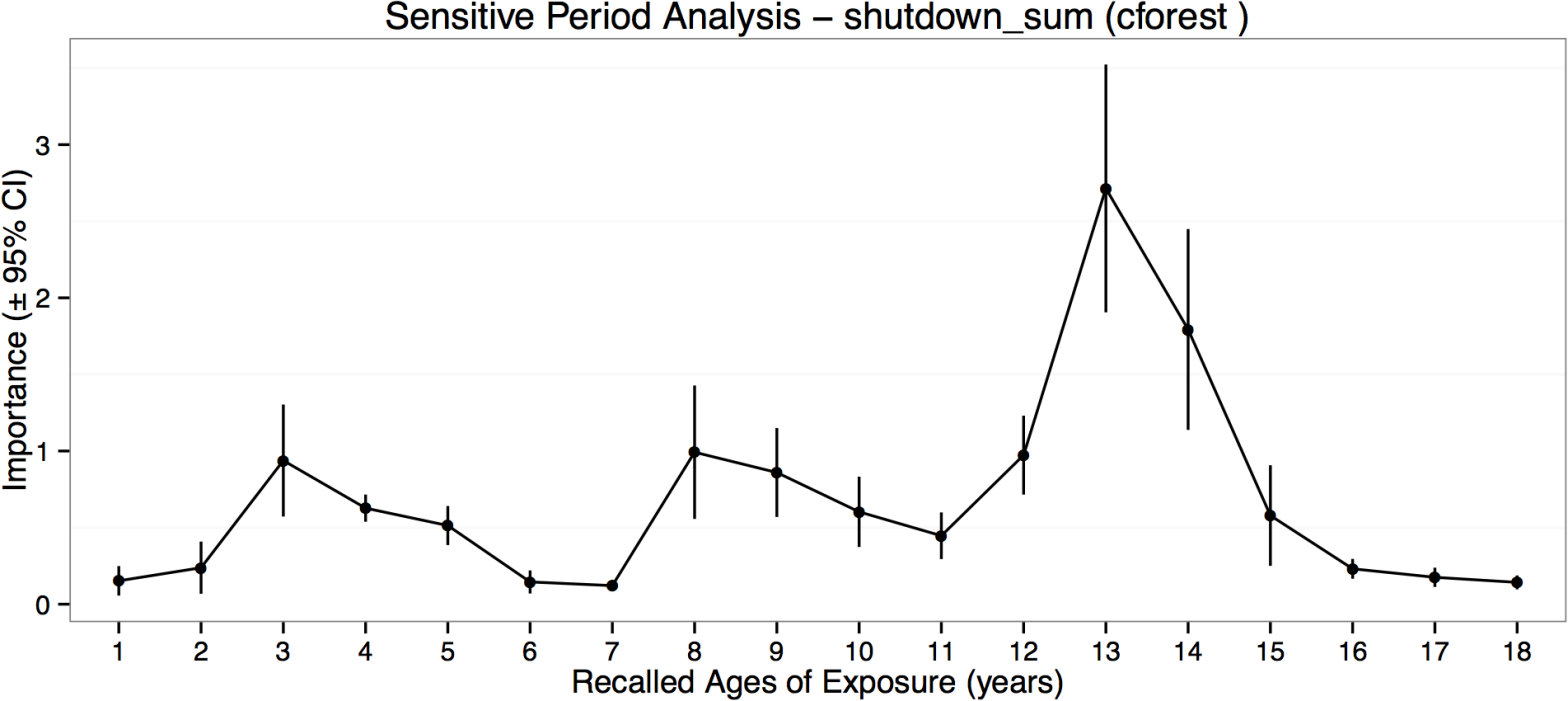
Timing of Childhood Adversities in Relation to Shutdown Dissociation. Maximal importance of exposure during each year of childhood in predictive accuracy for severity of shutdown dissociation regardless of type of abuse.

**Fig 5:**
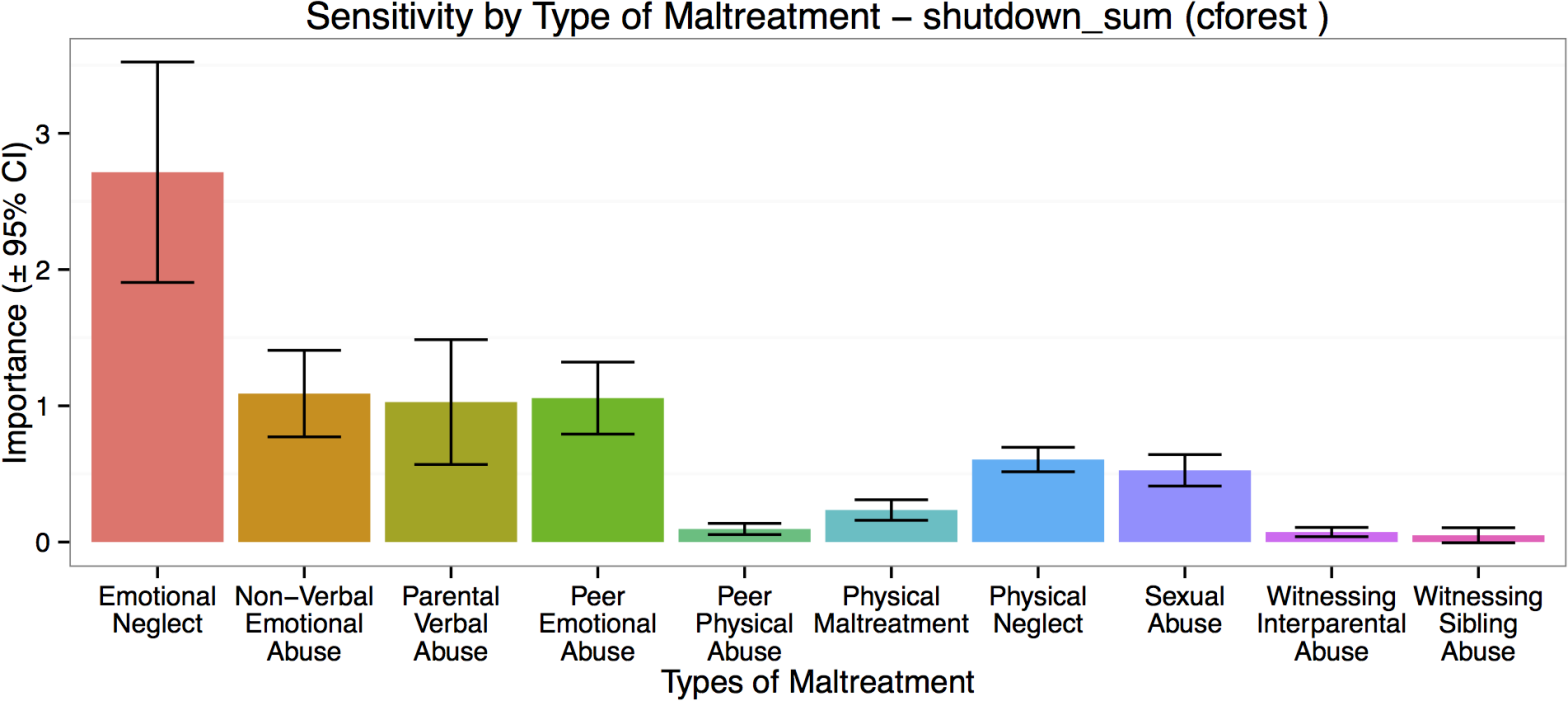
Type of Childhood Adversities in Relation to Shutdown Dissociation. Maximal importance of exposure to each type of maltreatment in predictive accuracy for severity of shutdown dissociation regardless of timing of abuse.

Predictive models derived from random forest regression with conditional trees fit the cross-validating sample well (mean *r* ^2^ = 0.31 ± 0.26, *p* < .003). The most important exposure ages were 12–14, 8–10 and 3–5 years of age, with peak vulnerability occurring at 13–14 years (Fig 4). Ninety-five percent confidence intervals indicated that importance of exposure at these ages exceeded importance of exposure at other ages. The most important type of exposure in the predictive model was emotional neglect followed by exposure to parental verbal abuse, parental non-verbal emotional abuse and peer emotional abuse, and to a lesser degree by physical neglect and sexual abuse (Fig 5). Witnessing interparental violence and violence toward siblings were the least important types of maltreatment in the predictive model.

### Sensitive Periods for Positive and Negative Symptoms

**Fig 6:**
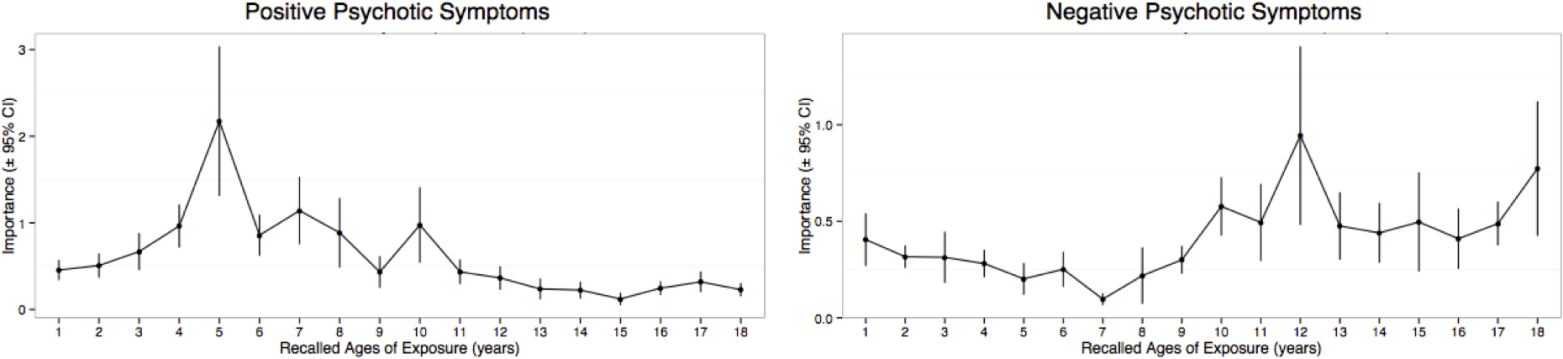
Timing of Childhood Adversities in Relation to Positive and Negative Symptoms. Maximal importance of exposure during each year of childhood in predictive accuracy for severity of positive and negative psychotic symptoms regardless of type of abuse.

Random forest regression with conditional trees was also used to assess whether there were sensitive periods when exposure to childhood maltreatment maximally increases risk for positive or negative psychotic symptoms. Severities of positive and negative symptoms were predictably modeled from the exposure data (positive symptoms *r* ^*2*^ = 0.19 ± 0.21, *p* < .020; negative symptoms *r* ^*2*^ = 0.20 ± 0.25, *p* < .030). Interestingly, it appears that maximum sensitivity to exposure for positive symptoms appeared to occur between 4–10 years of age whereas maximum sensitivity to development of negative symptoms appeared to occur between 10–18 years of age, with peak sensitivity at ages 5 and 12, respectively (Fig 6).

## Discussion

Consistent with previous studies, we found a high prevalence of childhood adversities in patients diagnosed with psychotic disorders e.g., [41,44]. More than two third of the sample fulfilled at least one or more criteria for adversities during childhood. In line with the literature, we replicate the relationship between shutdown dissociation and childhood adversities [7,8], even after controlling for additional traumatic stress in childhood and adulthood, the relationship between shutdown dissociation and adverse experiences in childhood remained significant. Both numbers of personally life-threatening events and witnessed event types during childhood showed positive associations with severity of shutdown dissociation in adulthood. Hence, it appears that witnessing life-threat to others may be sufficient to generate shutdown dissociation if it occurs during childhood. This finding supports the view that children may be more prone to dissociate when witnessing extreme threat to others, whereas adults may possesses the strength to encounter such threats with a fight/flight response [9,20]. If true, there are several possible explanations. First, for a young child an adaptive dissociative defense to reduce high anxiety and arousal may provide the greatest chance of survival when others are being threatened. Second, life threat to others, particularly parents or guardians, may be perceived by a child as personally life threatening, especially to the extent that they depend on these individuals for their survival [9]. We did not observe a relationship between different types of adulthood trauma and shutdown dissociation. One reason might be that once a dissociative responding style has been established during childhood due to adverse experiences and traumatic stress then re-victimization as well as exposure to further traumatic events may not significantly increase risk for shutdown dissociation.

Using the MACE we also provided a much more fine-grained analysis of the impact of specific types of childhood adversities on severity of shutdown dissociation. Interestingly, emotional neglect followed by exposure to parental verbal abuse, parental non-verbal emotional abuse and peer emotional abuse were most strongly related to symptoms of shutdown dissociation. This is in line with previous studies that empathized emotional neglect and abuse as a contributing factor for dissociative pathology in adulthood [8,34,35,36,38]. One explanation for the link between dissociation and emotional neglect could be that in a neglectful environment, dissociation may emerge as an automatic conditioned response to overwhelming emotions [73,74,75], and as a means of adapting to inattentive or unavailable caregivers. However in the long-term, this response pattern may interfere in integrative processes and representation of the self and environment, that results in fragmented cognitive, emotional as well as physiological processes [9].

On the basis of the neurodevelopmental model, we examined differential timing effects across the childhood. We found that preschool (ages 3–5), middle childhood (8–10), and peripubertal (12–14 years) periods were windows of vulnerability when exposure to childhood adversities was most important predictors of severity of shutdown dissociation in adulthood. In accordance with the result of a previous study, we could find sensitive periods for shutdown dissociations for adolescent stress [36]. Further very early life stress was an important predictor of ongoing shutdown symptoms in adulthood. It is also worth emphasizing the finding that early childhood stress was a key predictor of positive schizophrenic symptoms. Our observation that age 5 was the maximal stress-susceptible period predicting risk for delusions and hallucinations fits with observation of the importance of early exposure to maltreatment as a risk for psychotic symptoms as reported in a well-controlled prospective twin study [76].

It is tempting to speculate about the brain structures and function, which are stress-susceptible during these periods e.g., 3–5 years hippocampus formation, middle childhood for amygdala, and 14–16 years the frontal cortex [23,24,77].

Neuroanatomical abnormalities of hippocampal/ amygdala complex that varied with adverse experiences were found in patients with first-episode psychosis [78], as well its functional correlate for cognitive performance [79]. The interplay between the hippocampal complex, amygdala and the prefrontal cortex are important in the evaluation of incoming sensory information and in the regulation of arousal and emotions, which are processes interrupted by shutdown dissociation. Functional neuroimaging studies using idiographic trauma scripts suggest that there might be distinct neural circuits involving frontal and limbic regions that distinguish between PTSD patients with and without dissociative responses. Whereas patients with PTSD and predominantly hyperarousal symptoms displayed lower bilateral medial frontal activity and left anterior cingulate activity, the subgroup of patients with dissociative PTSD had increased right-sided medial frontal, medial prefrontal and anterior cingulate activity. These results were interpreted as emotional over-modulation in dissociative PTSD, versus emotional under-modulation in non-dissociative PTSD mediated by less intensive prefrontal inhibition of the limbic system [73]. Lanius and colleagues proposed that there may be two distinct pathways that underlie the finding of emotional dysregulation in patients with PTSD [74]. The first pathway describes emotional dysregulation as an outcome of fear conditioning through stress sensitization and kindling. The second pathway postulates an early neurodevelopmental vulnerability, often due to adverse childhood experiences, that produces difficulties in arousal and affect regulation, that may be further exacerbated following subsequent exposure to traumatic events resulting in PTSD. A similar concept was adapted to describe heterogeneous findings in patients with borderline personality disorder [75]. Similarly, a dissociative subtype has been proposed for schizophrenia [80] along with a duality model to explain the difference between dissociative and non-dissociative subtypes [4,81]. However, the emphasis on dissociative versus non-dissociative subtypes may be placing undue emphasis on symptomatology over etiology. Teicher and Samson [26] proposed instead that clinical syndromes be subtyped by presence or absence of childhood maltreatment, as the maltreated subtype appears to represent a clinically, neurobiologically and genetically distinct ecophenotypic variant. Subjects with this subtype will typically report more dissociative symptoms than subjects with the non-maltreated variant, but this is only one of several important differences.

Delineating sensitive exposure periods adds to our understanding of environmental and experiential influences on adult psychopathology. Subtyping schizophrenia by severity of dissociation or by maltreatment history may enhance clinical awareness of these potentially important differences, as they likely reflect significant underlying differences in brain structure and function, and may require distinct therapeutic approaches.

One significant limitation is the reliance on retrospective assessment of adverse experiences in childhood. Some question the reliability of such reports in general and are even more suspicious of the veracity and reliability of retrospective self-report in patients with severe mental illness. In a comprehensive review Brewin and colleagues [82], found scant evidence to support these concerns. What we know in comparing retrospective to prospective reports is that adults tend to minimize their degree of exposure on retrospective report [83,84]. Hence retrospective exposure rates are lower than prospective rates suggesting a problem with false negative reports but not false positive reports. Individuals reporting abuse retrospectively were those who typically endured the most severe abuse on prospective assessment [83]. This fits with other studies showing that adult reports of abuse are verifiable [85]. Previous studies provide specific evidence of sufficient test-retest reliability even in patients with severe mental illness [86,87]. Indeed, self-reported exposure to maltreatment has been found to be highly consistent over years in psychotic individuals and not significantly influenced by the severity of their psychosis or their depressive symptoms [88].

## Conclusions

Our results suggest two major pathways to dissociative pathology. First, dissociative responding could be understood as a useful adaption to facilitate survival during life-threat that may reactivate whenever the fear network is triggered [11,89]. Second, it may develop during childhood as an adaptive response to an emotionally neglectful and emotionally abusive environment. Such an environment may poorly prepare an individual to regulate or cope with intense emotions, leading to episodes of shutdown whenever their limited regulatory capacity is overwhelmed. Identification of sensitive periods in the development could help to yield insights into potential disturbance through stress and long-term consequences. The timing of early life stress may contribute as well for the striking heterogeneity of behavioral, emotional and cognitive psychopathology across disorders [26]. Whether these results are specific for patients with psychotic spectrum disorders, and how these results of sensitive periods are based on structural or functional neurobiological differences require future research.

## Supporting Information

S1 Dataset Dataset. (XLSX)
